# Selections

**Published:** 1816-06

**Authors:** 


### PART III.

### SELECTIONS.

### FRENCH DICTIONARY OF MEDICAL SCIENCES.

( Continued from p. 359. )

*Article Cadavre.** (English, *dead body*, *corpse*; Greek, *πτωμα*; Latin, *cadaver;* German, *todter korper-aas;* Italian, *cadavero*) We have here to consider the subject under its relations with forensic medicine.

It was not until the sixteenth century that dead bodies were submitted to medico-legal investigation. The horror which human dissections inspired in the ancients, and the religious awe with which they beheld the mortal wreck of their fellows, not only retarded the progress of anatomy and physiology, but excluded the light of medicine from the researches of the forum. The Jewish law, indeed, condemned to capital punishment that person who

---

* Circumstances of a most impressive nature, which, however, it would be improper at present to detail, have induced us to select this article for our subject of discussion. That the medico-legal examination of bodies shares the general neglect experienced by every branch of forensic medicine, in this island, is a truth lamentable as notorious. Not a year passes without producing numerous instances of this kind. To no inability or wilful negligence on the part of professional men is the evil attributable; for never did country contain a body of medical practitioners more benevolent, conscientious, and enlightened, than our own at present boasts. But it is natural enough, that they should cultivate with a lukewarm spirit, and attach little importance to a branch of knowledge, not much consulted or relied upon in British courts of Justice. Few days only have passed, since we were told, by a man of high legal celebrity, that nothing like demonstration could be introduced into questions of forensic medicine: in fact, that all medical evidence on these occasions was mere matter of opinion! There exists in this part of our generally admirable system of legislation, a radical defect which loudly calls for notice and redress.—EDITORS.

inflicted upon another a blow followed by death; but there is reason to believe, that the application of this law was determined by the phænomena characterizing cessation of life, and not by anatomical inspection. The opinion of the physician, Antistius, that only one of the twenty-three wounds received by Julius Cæsar was mortal, rested upon external examination: that wound had penetrated into the thorax. Many other examples might be cited, to prove that the inspection of bodies was confined to superstitious practices. In their trials for homicide, means of proof, the most absurd, were preferred to the evidence which dissection might have afforded. Thus, for example, the bleeding of a corpse on the approach of the murderer was one of the principal phænomena upon which they depended for discovery of the truth.*

The medico-legal examination of bodies was once exclusively confined in France to a limited number of physicians and surgeons, *called officiers de médecine du barreau* (officers of forensic medicine.) By the existing laws, the graduates of any faculty of medicine in the French empire are allowed to perform this office.†

I. *Rules to be observed previously to the examination of a body.* In France, it is necessary that the physician employed in this office, should receive the requisition from persons properly qualified; and that all who attend him as assistants or witnesses, should possess the titles specified by the law.

*Of the external circumstances relative to the state in which the body is found.* Such, for example, are the hour and place in which it has been discovered; its posture; whether it were cloathed, covered, how and with what; whether in contact with any substance capable of exercising an influence upon it, and especially of accelerating or retarding the progress of putrefaction. Here the temperature, dryness, or humidity of the surrounding bodies, and season of the year, should be accurately noted. If any deadly weapon be found near the deceased, its position, with respect to the body, should be accurately examined before removing it; and no detail omitted, which, however trifling in appearance, may eventually prove important, particularly in cases where suicide is to be distinguished from assassination.‡ This part of the business, it is

* This preposterous superstition still prevails in some parts of England. We have recently observed an example of its influence.—Editors.

† Some such regulation is surely wanting in this country. We have actually known a man, styling himself a surgeon, allowed to inspect a body and give evidence, in a case of poisoning; who confessed, on cross examination, that he had but *once* in the course of his life *seen* a human subject opened!—Edit.

‡ Some years since, a French prisoner of war was shot in a solitary place, distant about one mile from our residence. We were among the first who reached the scene of blood. A report had got abroad, that he had fallen in a quarrel with one of his countrymen; hence our office demanded especial accuracy and circumspection. The body, completely lifeless, lay stretched on the back: a pistol, which bore evident marks of true, commonly devolves on the civil officer, and is regarded as his province; but, why should not the juridical physician assist in researches which should be common to both, inasmuch as they relate to the body of the deceased, and are alike directed to one object?

*Of the removal of the body, and choice of a place for inspection.* Examination of the body cannot often be made on the place of discovery. In this case, the juridical physician should himself preside at the removal, and take the necessary measures to protect the body from the slightest injury during conveyance. For this purpose, a hand-barrow is always preferable to a cart: when the latter only can be obtained, it should be well spread with straw, and proceed slowly. Under these circumstances, particularly when there exists a suspicion of poison, all the orifices should be closed, by which any matters, about to become the subject of chemical analysis may escape. The head also should be supported to prevent undue motion; nor ought the medical attendant to quit the vehicle for a moment. Whenever dissection cannot be performed on the spot where the body is found, its external condition should always be examined previously to removal; so that, if any accident befall on the way, the consequences of it may not be attributed to the original injury.

The place chosen for the inspection of the body should be well lighted and airy. Every precaution should be taken to guard those around from noxious consequences: if the body exhale any offensive smell, fumigations, and aspersions of vinegar are to be employed. All idle persons should be excluded.

*Of the period of examination.* This should never take place on the approach of night, or by candle-light. Morning is, on all accounts, to be preferred.

*Of the instruments requisite for the operation.* These are a case of common dissecting instruments; several saws, (*for inspection of the brain is, in all cases, an essential point*) sponges, flexible probes, tubes for inflation, a large tin or copper syringe, with variously-sized pipes; a coloured liquid, (not red) with which to explore the nature of any vascular lesion; a measure of length; compasses; scales and weights; and, lastly, a large graduated glass measure,

---

having been recently discharged, was firmly grasped in the right hand. The ball had entered the roof of the mouth, about the junction of the soft and bony palates, taken a course upward and backward; and, having traversed the cerebellum and occipital bone, lodged beneath the cranial integuments. The cheeks and lips were uninjured; no teeth fractured or displaced. A countryman, walking near the spot, had seen the flash, and heard the report, of *one* pistol only. Considering the exemption of the face and teeth from injury, we gave it as our opinion, that the pistol must have been fired while the mouth was widely opened. The direction of the wound obviously confirmed the inference thus drawn, that it could only have been inflicted by the hand of the unfortunate captive himself. —EDITORS.

II. *Rules to be observed during the examination of a body.* In general, the inspection should not be undertaken until life has been extinct twenty-four hours; unless the nature of the injury be such as to exclude all suspicion of dormant vitality, or its immediate performance be enjoined by legal authority.*

A question has frequently been agitated, whether a body, when attacked by putrefaction, should be submitted to juridical investigation? In Russia, this question has been negatively resolved; inasmuch as putridity renders examination inconclusive. But this lution is as inexact as the question from which it has arisen; for, in truth, internal examination of a body should never be deemed impracticable, until putrefaction be so far advanced as to deform the organs, and render their lesions indistinct: and again, lesions may exist upon parts inaccessible to putrefaction, as the cranial bones. It may be asserted, that, in all cases, commencement of animal decomposition is no obstacle to anatomical research. Secondly, it has been asked, whether, when a body is so mutilated or disfigured as to render hopeless the discovery of the cause of death, examination should be persisted in. The answer to this question is negative; because such mutilation may possibly be inflicted to mask the real cause of death. Thus, the remains of a person destroyed by poison may be intentionally dismembered by an artful assasin. Or let us suppose a lonely cottage, inhabited by a man and wife. The dwelling is destroyed by fire, and the bodies of the unfortunate pair discovered amid the ruins. The magistrate, accompanied by medical practitioners, repairs to the spot. The bodies are examined and betray unequivocal marks of injury inflicted by fire arms, or perhaps even the bullets themselves are discovered. Thus suspicions are excited, which lead to the detection and punishment of the murderers.

Previously to opening the body, the surface should be minutely examined, and all the blood or dirt, with which it may be soiled, cleared away. The length of it should then be exactly ascertained: in short, the description should be traced without omitting the slightest scar or congenital mark. Attention, particularly rigorous, should be exercised in cases of suspected child-murder. The inspection of the surface should extend even to parts the most secret and retired: the cavities of the mouth and nose; nucha, ears, fontanelles, arm-pits, those parts of the thorax (in the female) covered by flaccid and pendulous mammæ, sexual organs, and anus. This precaution is requisite, even when evident lesions exist, adequate, of themselves, to explain death. In illustration of this precept, it will suffice to observe, that there may have existed between the victim and *his* murderer connexions which such research will sometimes elucidate. Humanity may be spared the shame of indicating their nature.

---

* Beyond the term of twenty-four hours, examination, if practicable, should never be delayed.—Edit.

It is highly important to specify the situation, form, extent, and direction of external injuries. Wounds of all kinds especially require their dimensions to be determined; and alteration of them, by an aukward method of examining, must be avoided. It is always best to investigate these points previously to inspection of the supposed instrument of death, in order that the mind may be kept clear from dangerous prejudices; for it is well known, that changes both in the length and breadth of wounds take place after death. To establish precisely the nature of these changes would require a series of experiments not yet made. All ecchymoses from an internal cause should be cautiously distinguished from those consequent on external violence.

From the moment when dissection commences, the attention of the juridical physician should be redoubled; for it is then that if wounds exist, he is to establish, by observation of their depth, extent, and direction, not only the nature of the instrument which has inflicted them, and the relations of its dimensions with those of the wound, but the precise seat of injury. It should also be specified whether the wound were made with a cutting, bruising, or puncturing instrument; whether by an ignited or corrosive substance; what are the vessels, nerves, viscera, muscles or bones, injured;* whether any foreign body were discovered in the wound; whether there existed inflammation, suppuration, gangrene, effusion, dislocation, fracture, or rupture; and, finally, in what manner the neighbouring parts were affected. Each species of death will, moreover, demand particular precautions, exclusively applicable to it.

It was enacted by an ancient (French) law, that, in all such cases, the three principal cavities of the body should be examined; yet this has been neglected, whenever the appearances displayed by any one cavity have been supposed to indicate an efficient cause of death. *But such omission ought never to be tolerated*; since it may unexpectedly lead to fatal consequences. *Thus, two surgeons, from having neglected to open the head of Jean Chassagneux, of Montbrisson, who died from apoplexy during a scuffle with his son and daughter-in-law, caused the unhappy persons to be condemned as parricides: and they had unquestionably perished on a scaffold, but for the interference of the celebrated Louis, who demonstrated the insufficiency of the examination, and innocence of the accused.*† Reader, mark this!

---

* The importance of these minute researches, and their application to practice, we shall, ere long, have occasion to illustrate somewhat diffusely.—Editors.

† Facts like these can never be too deeply imprinted on the recollection of all who are interested in deciding, or giving medical evidence, upon questionable cases of murder; and we think that no person, accused of this crime, should be condemned *upon purely circumstantial evidence*, until the most minute investigation has been instituted into the previous

III. *Rules to be observed after the examination of a body.* The inspection finished, all the parts should be carefully replaced, and the cavities filled with saw-dust or ashes, to prevent the escape of blood or other fluids during interment. The body should then be closed in the most neat and decent manner. This is the time for the practitioner to revise the notes made during dissection, and to rectify any dubious or inaccurate expressions: nor ought he to deliver his conclusions upon this report until he has reflected upon it in retirement, and his mind has been tranquillized by a night's repose. He should, moreover, especially abstain from answering the questions of an inquisitive mob. Nothing is more dangerous in criminal affairs than public clamour: and it is impossible to appreciate the consequences to which, on such an occasion, the unguarded assertion of a juridical physician may lead.*

The principal monographs on the medico-legal examination of bodies are, those of *Libavius*, 8vo. Frankfort, 1594; *Pietre*, fol. Paris, 1634; *Kirchmayer*, 4to. Wittenberg, 1669; *Feltman*, 4to. Groningen, 1673; *Bodin*, 4to. Halle, 1703; *Emmerich*, 4to. Konigsberg, 1710; *Leyser*, 4to. Helmstadt, 1723; *Alberti*, 4to. Halle, 1726; *Detharding*, 4to. Rostock, 1726; *Mauchart*, 4to. Tubingen, 1736; *Gericke*, 4to. Helmstadt, 1737; another, 4to. 1738; *Westherof*, 4to. Leyden, 1738; *Teichmeyer*, 4to. Jena, 1742; *Boehmer*, 4to. Halle, 1747; *Heister*, 4to. Helmstadt, 1748; *Fabricius*, 4to. Helmstadt, 1750; *Schoenmetzel*, 4to. Heidelberg, 1766; *Lieberkuehn*, 4to. Halle, 1771; *Roose*, 8vo. Bremen, 1800—third edition (posthumous), Frankfort-on-the-Maine, 1804—fourth edition, revised by *Himly*, Frankfort, 1811 (translated into French, and enriched with notes and two original memoirs, by *Marc*, 8vo. Paris, 1808); *Occhy*, 8vo. Prague, 1802; *Crusius*, 8vo. Gottingen, 1806: *Baetens*, 4to. Paris, 1808.

Several other detached treatises might be enumerated, but the preceding list will suffice. We, at this moment, recollect no Eng-

---

moral and physical condition of the deceased, and the prevalent diseases of his family; and until *every* cavity of his body has been explored by patient dissection. What would be the feelings of all concerned in the condemnation of a supposed criminal, if, after execution, it should be discovered, that the person, on whose account he suffered, had perished from spontaneous apoplexy, rupture of the heart or a large artery, bursting of a chronic abscess, or other cause unconnected with external violence! The history, cited in our Selection department for March last, affords a most instructive lesson. We cannot quit this momentous subject without again earnestly calling the attention of medical men to it.—EDITORS.

* The whole of this paragraph, particularly its concluding part, contains excellent advice. We recollect an instance, in which the inconsiderate assertion of a surgeon, on momentary inspection of the body of a young man who had died in a mysterious manner, produced on the public mind an influence highly prejudicial to the character of two *innocent* persons, and endangered, for a time, their personal safety.—EDITORS.

lish work in which the subject is comprehensively discussed.*—EDITORS.

### FOREIGN JOURNALS.

### "*History of a Case of Hydrophobia, which terminated fatally, after an Hour and a Half's Treatment in the Charité Hospital (at Berlin); with the Appearances on Dissection.*†

" ON the morning of October 30th, 1814, between the hours of eight and ten, Gottfried V. a journeyman butcher, aged 29, was brought into the hospital. Four weeks previously, he had been bitten in the hand by a mad dog, and died in an hour and a half after his admission. The widow, in whose service he lived, kept a large dog, which was commonly chained up, but sometimes let loose. From the testimony of several police officers, it appeared that this animal was actually mad; that he wandered about, and must have bitten many other dogs. Where, when, and by what rabid animal he had himself been bitten, we were unable to ascertain. No sooner was his malady observed, than he was, on all sides, pursued. Upon being hard pressed in the city, he rushed into the Spree, and again made land on the opposite shore of the river. Gottfried V. who met with him here, enticed him to approach; and, as soon as he could get possession of him, took him up. The people were rejoiced to see the dog secured; and, while V. held him, proceeded to knock him on the head. Ere this, however, could be accomplished, the furious animal sprang in his own defence, and bit V. in the right hand, between the thumb and fore-finger. V. rejected the medicine and treatment recommended to him by a person in the neighbourhood; but employed instead a superstitious remedy. What external application was used to the hand is not correctly known; but certain it is, that the wound was completely healed within eight days; and that the patient, during and after this time, found himself well, although he sometimes harboured apprehensions of his danger.

" On Friday, October 28th, his first complaints began. He felt rending pains in the cicatrix of the wound, amounting even to agony; there was great heat and much thirst; and he found him-

* The sensible preface to Dr. Male's *Epitome* does not exactly come under this description; but it may be read with great advantage.—EDIT.

† Anxious to accumulate all possible evidence on the treatment of hydrophobia by blood-letting, we have extracted this case from Hufeland's *Journal der Practischen Heilkunde;* not aware that it has appeared in any contemporary Journal. No decisive inference, however, should be drawn from the unfortunate termination of this case; as venæsection was not employed till a period at which no human resources could avail; and the same remedy has since been crowned with complete success in a case of hydrophobia on the Continent.—EDITORS.

self so exhausted and ill, that he was obliged to lie down on the bed. All the symptoms of a violent continued fever were rapidly developed. The patient from hour to hour was restless; fell into the most vehement agony; began to spit copiously, and was no longer able to swallow liquids. From the report of a friend, who accompanied the patient to the institution, it appeared, that a physician had been consulted. He mistook the disease for a mere cold: we know not what remedies were prescribed by him. The hydrophobic symptoms, nevertheless, were still farther developed; and, during the night of the 29th, a decided abhorrence of water, and all other fluids, had taken place. The spitting continued incessantly: the patient complained, and moaned piteously, without being able accurately to describe his sensations. The neck was dry, and the extremities were affected with fever and spasms.

"As soon as the patient had reached the hospital, I was summoned to him; and found his situation marked by the following appearances: His body was of the middle size, strong, muscular, and very well fed. His whole frame trembled, particularly the lower limbs; the muscles of which were constantly drawn by spasms, so that he could not hold them still a moment, even when sitting down. The expression of his countenance, and his whole demeanour, were completely different from those of the hydrophobic patient who died here in August. Mental disorder and confusion in that case existed, even to the wildest phrenzy: here, on the other hand, was the deepest shade of melancholy, attended, however, with perfect recollection of all which had passed, and with consciousness of his desperate situation. His appearance bespoke the deepest and most acute suffering, so that no one could behold him without heart-felt sympathy. He seemed to listen, when any one consoled him with the assurance that he might yet recover: but the expression of misery and sense of pain were not thereby diminished. With difficulty, an answer was got from him; which was, that *he could speak but little, for his tongue was become hard.* He spoke in a confused and inarticulate manner; so that he constantly spat out the saliva collected in quantity in his mouth. He put out his tongue trembling and covered with saliva, which, every moment, flowed copiously afresh. The complexion was dirty; the lips red and tremulous; the eyes staring; the pupils dilated, and the facial muscles convulsed. The pulse was uncommonly hard, moderately full, and about 140 in a minute. In the case which happened in August, the arteries also beat exceedingly hard, but far less frequent than in this case. Shining substances, as glass, gold, watches, and even liquids themselves, when held up to him, he could look upon without irritation; yet he refused to drink water. When any one exhorted him in a friendly manner to taste it, he strove constantly against it; yet, at last, yielded to the invitation. He could not, however, in spite of all his efforts, swallow it, and rejected that which he had taken into his mouth with violent straining. The carotid arteries pulsated vehemently, and the veins of the hands and arms were exceedingly turgid with blood.

"Full blood-letting, to the commencement of syncope, was apparently the treatment indicated in a strong and plethoric subject, in the meridian of life, upon whom no evacuating remedy, neither venæsection nor leeches, had been employed; yet was it, in this case, to be regretted, that the time, from the evening of the 28th to the morning of the 30th of October, had passed away so utterly unimproved; wherefore it was to be feared, that this, like every other remedy, would come too late. Ere the operation was performed, the most violent spasms of the throat, face, and whole body, came on: indeed, the whole muscular system was in a state of convulsion. I immediately directed a large orifice to be made, first, in the right arm; and then, as the flow of blood from this ceased, in the left: whereby I might observe, myself, the variations which took place in the pulse as well as in the whole body. Although the orifice was large, and the blood flowed in a good stream, yet was no effect perceptible, either on the system or the pulse, even when much blood had been lost. The convulsions, the constant ejection of saliva, the loud groaning and sighing, the inexpressible sadness of his countenance, the condition of his eyes, and hardness and rapidity of his pulse, were not perceptibly altered. When the first traces of a slight inclination to faintness appeared, as several pounds of blood had already been taken, I directed the vein to be closed, and actual syncope speedily ensued. The patient revived on irritating his nostrils; and began again, as before, to spit and groan violently. Thus the treatment was without effect, and soon its ill success became apparent: he continued to spit, tremble, and conduct himself strangely to the last moment.

"The blood which had been drawn quickly formed a somewhat firm coagulum, was of a dark red colour, and shewed no inflammatory crust.

"Soon after death, the body was laid in a prone position, with the view of preventing the gravitation of the blood, and congestion of the deep-seated viscera. Twenty six hours afterwards, it was opened in the presence of several professional gentlemen." (We think it needless to specify their names and titles.)

"Upon external examination of the body, we observed a number of blue spots, particularly in those parts upon which it had lain.

"After the cranial cavity was opened, we found the vessels of the dura mater somewhat distended with blood; yet could not this state be termed inflammation. The structure and hue of the brain and cerebellum, the pia and dura mater, the plexus choroides, and ventricles, displayed no remarkable deviation from the natural state; except that the vessels were every where filled with thin fluid blood.

"The internal membrane of the pharynx was somewhat red and slightly inflamed. In the œsophagus, as far as the cardia, no inflammation was observed; but the tube, in this part, was of considerable strength, and in many places, as dark red as in incipient gangrene. At the pyloric orifice of the stomach, no inflammation existed: yet the intestines, the stomach, particularly its fundus, and several parts of the ileum, displayed it in a marked degree. The inner surface of the larynx was, in several places, redder than natural; and yet more the trachea and bronchia; the internal membrane of which was of a very deep red, and highly inflamed. The lungs contained in their vascular structure a quantity of black blood, which gushed out copiously on each incision. In the ventricles of the heart also, particularly the right, there was a quantity of thick and coagulated blood. The viscera of the abdomen, the liver, kidnies, pancreas, and spleen, displayed no morbid appearance. The bladder was distended by a quantity of strong and fœtid urine. In the nerves of the neck, the great sympathetic, vagi, and recurrent, no visible change, or inflammation, had taken place. The large vessels were equally exempt. The muscular flesh was of a very dark red colour; and the body, upon being opened, exhaled a penetrating smell, very much like that of putrid game; although the deceased had yet lain but twenty-six hours."—" HORN."

*Dropsy of the maxillary sinus.** " In the early part of April, 1814, the author met a young man, whose appearance was rendered disgusting by a deformity consequent on excessive dilatation of the right maxillary sinus. The individual in question, aged 19, had always enjoyed good health. Five years previously to this period, his head had been slightly wounded in several places by a fall; but the cheek had sustained no perceptible injury. Yet, in the following year, this part seemed to project more than the other, and continued to increase without causing pain. The sinus, at length, became extremely dilated: its parietes, pushed out in every direction except towards the eye, yielded to the pressure of the finger a noise resembling that of a somewhat hard skin of parchment. The nasal septum, and nose itself, were driven upon the left cheek: the portion of palatine vault, on the affected side, yielded also to the pressure of the finger. The teeth had undergone no change.

" In passing the finger along the lip and cheek, a little above the summit of the alveoli, a crack of the inferior part of the external wall of the sinus was readily discovered: and in this point there was a decided fluctuation; whether this arose from the presence of a fluid, or of a soft polypus, it was not easy to determine.

" The patient having been properly seated, the sinus was opened by an incision made with a bistoury, along the alveolar margin, and upon the softened part of the bone. Immediately, a *limpid, inodorous, colourless fluid* was seen to escape; in fact, according to M. Sauvé, the product of a pure and simple dropsy.

* By Dr. Sauvé, Lorient; commented on by M. Ribes. *Bulletin de la Faculté de Médecine de Paris, &c.* 1816. No. I.

Two lateral and perpendicular incisions were made, three quarters of an inch from each other, by means of scissars. A superior incision, parallel to the first, removed a square, formed by a portion of the raised border of the maxillary bone. By this opening, the interior of the sinus was exposed. Its membrane was somewhat discoloured: no pain was excited by touching it with the finger. After having been injected with warm wine, the tumefied osseous parts were pushed back; and their flexility allowed the face and palatine vault to be restored to their original form. The same injections were continued during fifteen days. Neither pain nor tumefaction ensued; but when the patient was dismissed, he had yet a large opening in the maxillary bone."

The preceding case, though extraordinary, is not unique; since, as M. Ribes observes, analogous histories may be found recorded by Deschamps, in his *Dissertation on the Diseases of the Nasal Fossæ and Sinuses;* by Runge, in the first volume of *Haller's Surgical Dissertations;* and by Fauchard. Both the latter are cited by Bordenave, in his *Memoir on the Diseases of the Maxillary Sinus;* and Professor Lallement assures M. Ribes, that he has himself seen an example of it in a female.

We shall not attempt to follow M. Sauvé in his hypothesis, to explain the formation of a serous fluid by the vessels of a mucus membrane; since we are very much inclined to adopt the conclusions drawn by M. Ribes, that the fluid in question, notwithstanding its physical characters, was not serum, but the mucous of the nasal cavities. "In truth," adds M. Ribes, "the action of cold, by increasing the secretion of the nasal fluid, furnishes it in all its purity. It is then clear, without smell, and limpid as distilled water. The contact of oxy-muriatic gas (chlorine) produces a similar effect on the pituitary membrane; and it was the mucus secreted under the influence of these causes, which served Vauquelin for the purpose of analysis: yet the Professor does not say, that the nature of the fluid, in this state of limpidity, had suffered any change."—EDITORS.

